# “¿Qué opción les queda a las personas, más que nosotros?”: Las funciones de los consultorios adyacentes a farmacias en la pandemia de covid-19

**DOI:** 10.18294/SC.2023.4280

**Published:** 2023-03-16

**Authors:** Ana Victoria Morán Pérez

**Affiliations:** 1 Doctora en Antropología. Profesora, Escuela Nacional de Antropología e Historia, Ciudad de México, México. ana00.moran@gmail.com Escuela Nacional de Antropología e Historia Escuela Nacional de Antropología e Historia Ciudad de México México ana00.moran@gmail.com

**Keywords:** COVID-19, Pandemia, Atención Médica, Sector Privado, Percepción del Riesgo, COVID-19, Pandemic, Medical Care, Private Sector, Risk Perception

## Abstract

Con la llegada del coronavirus a México, los consultorios adyacentes a farmacias desempeñaron un rol preponderante en el diagnóstico, atención y prevención del covid-19. De acuerdo a las encuestas nacionales, entre el 11,7% y el 23% de las personas con síntomas de covid-19 se atendieron en uno. Por ello, este artículo busca identificar el papel de los consultorios adyacentes a farmacias (CAF) como sistema de salud privado que atendió a personas con síntomas de covid-19 en la ciudad de Oaxaca y describir y analizar los factores que influyeron en su utilización. Desde una metodología cualitativa, entre septiembre de 2020 y agosto de 2022 se entrevistó a 12 médicos y médicas y se aplicó un cuestionario a 59 personas usuarias de los consultorios adyacentes a farmacias del municipio de Oaxaca de Juárez. Asimismo se hizo una recopilación y análisis de fuentes secundarias. Entre los hallazgos, se describen sus funciones como frente de atención al covid-19 y a otras necesidades de salud que emergieron con la crisis sanitaria y se analizan los factores determinantes en las trayectorias de atención de personas usuarias de estos consultorios, como son el incremento en la percepción del riesgo y la desconfianza hacia los servicios públicos o hacia las estrategias implementadas por el gobierno federal.

## Abstract (en)

With the arrival of coronavirus in Mexico, doctors’ offices adjacent to private pharmacies (DAPPs) played a major role in the diagnosis, care, and prevention of Covid-19, providing treatment for 11.7% to 23% of people with Covid-19 symptoms according to national surveys. Therefore, this article seeks to identify the role of DAPPs as a private health system providing care for patients with Covid-19 symptoms in the city of Oaxaca, and to describe and analyze the factors that influenced their utilization. Using a qualitative methodology, twelve physicians were interviewed and 59 users responded to a questionnaire at doctors’ offices adjacent to pharmacies in the municipality of Oaxaca de Juárez between September 2020 and August 2022. Secondary data were also collected. Among the findings, the function of these offices at the front line of care for Covid-19 and other health needs that emerged with the public health crisis is described, and the determining factors in care trajectories of users that sought care there are analyzed, such as the increase in perception of risk and mistrust towards public services or strategies implemented by the federal government..

## INTRODUCCIÓN

Las pandemias muestran de forma cruel cómo el capitalismo neoliberal incapacitó al Estado para responder a las emergencias. Boaventura de Sousa Santos^1^

Desde su inicio, la pandemia de covid-19 desveló una serie de contradicciones y precariedades de las sociedades actuales, tal como lo plantea Eduardo Menéndez al señalar que los procesos de salud-enfermedad-atención-prevención son la puerta de entrada para mirar otros procesos de carácter económico-políticos e ideológico-culturales que forman parte de la vida social^2^. Bajo esa premisa, el coronavirus contribuyó al redescubrimiento, o quizá más bien a la confirmación, de una serie de problemáticas emergentes del modelo neoliberal. Una de las más visibles refiere a la incapacidad de los sistemas sanitarios para hacer frente a una situación de estas dimensiones.

En el caso de México, se expuso la crisis de un sistema de salud que, a consecuencia de la implementación de las reformas neoliberales en las décadas de 1980 y 1990, presenta una notoria desigualdad en el acceso a servicios de salud; problemas de cobertura de atención, sobredemanda y saturación; precarias condiciones laborales de los trabajadores sanitarios; crisis de infraestructura y equipamiento de los servicios; problemas de desabasto de medicamentos en los tres niveles de atención, así como la presencia de un aparato burocrático que ralentiza los trámites administrativos^3^.

Además, esta emergencia sanitaria llegó en un momento crucial de ajustes del sistema de salud, dado que después de diecisiete años de operación del Seguro Popular, el 1 de enero de 2020, unos meses antes de llegar el SARS-Cov-2 a México, fue reemplazado por el Instituto de Salud para el Bienestar (INSABI), planeado con el fin de operar de manera directa los sistemas de salud que estaban a cargo de los gobiernos locales financiados por el Seguro Popular, asegurando la atención a la población no derechohabiente^4^. Sin embargo, su instauración se vio parcialmente truncada por la pandemia^5^. Dos años después de comenzar a funcionar, no logró cumplir con sus metas, por lo que en agosto de 2022 se anunció la creación de un nuevo organismo público descentralizado: el Servicios de Salud del Instituto Mexicano del Seguro Social para el Bienestar (IMSS-Bienestar)^6^. Por ende, el covid-19 arriba en un momento complejo y de transición del sistema de salud, lo cual dificulta su capacidad de respuesta al problema sanitario.

En este momento crítico, adquieren relevancia otros actores del sistema de salud mexicano, como son los prestadores de servicios de salud privados, los cuales terminan de asentarse como recursos primordiales para atender las necesidades de salud de amplios sectores poblacionales. Esta tendencia ya venía asomándose en los últimos años, producto no solo de las políticas de privatización de la salud propias del esquema neoliberal, sino también de la insuficiencia de los servicios de salud públicos para atender al grueso de la población y de la desconfianza e inconformidad de las personas usuarias hacia ellos.

En las últimas décadas, se incrementó la oferta y demanda de servicios de salud privados en todo el país. De acuerdo con un estudio realizado por González Block *et al*.^7^, el sector privado ofrece el 44% del total de las consultas ambulatorias y el 22% de los egresos hospitalarios. No obstante, una de las características centrales del sector privado en México es su segmentación y heterogeneidad, lo cual se manifiesta en los recursos, infraestructura, costos de los servicios ofrecidos, y en la población a la cual van dirigidos. 

La privatización de la salud se expresa de formas distintas en función de su financiación, organización/gestión y provisión de la atención^8^. De tal modo, el sector privado se constituye tanto por corporativos hospitalarios que brindan atención especializada a población de la clase media y alta; por clínicas o pequeños hospitales de “barrio”, que ofrecen servicios de segundo nivel de atención, a los que acuden los sectores populares; o bien, por consultorios médicos privados y consultorios adyacentes a farmacias (CAF) que brindan atención ambulatoria y a los cuales acuden sectores variados. Estos últimos se conforman como un servicio que se financia a través del pago directo del usuario, y se gestiona directamente por empresas privadas, las cuales proveen los servicios médicos a partir de la asociación entre consultorio y farmacia.

La Encuesta Nacional de Salud y Nutrición 2021 (Ensanut) sobre covid-19 indica que de una submuestra de 3.027 individuos que reportaron haber tenido una necesidad en salud y recibir atención médica, el 56% acudió a servicios de salud privados^9^. A su vez, el presidente de la Asociación Nacional de Farmacias Mexicanas (Anafarmex) señaló que, en 2018, alrededor de la mitad de la población utilizó servicios privados, proporción que creció para 2020^10^. Dentro de los servicios de salud privados, los CAF tuvieron un lugar central, como se pretende describir en este artículo, en la medida que cubrieron un vacío del sistema de salud que se agudizó con el coronavirus. En la Ensanut 2021 se muestra que el 11,7% de las personas diagnosticadas como positivas a covid-19 y que recibieron atención médica, lo hicieron en un CAF^9^. Sin embargo, es probable que este porcentaje presente un subregistro, debido a que la opción indicada en el cuestionario, consultorio dependiente de farmacia, es un término técnico con el cual parte de la población puede no estar familiarizada^11^. Por ello, es posible pensar que pese a haberse atendido en un CAF, algunos entrevistados reportaron atenderse con un médico privado.

El presente artículo presenta resultados parciales de una investigación que buscó responder a las siguientes preguntas: ¿cuáles fueron las funciones desempeñadas por un servicio de salud privado como los CAF en el marco de la pandemia y en el contexto de Oaxaca de Juárez? ¿De qué modo influyen la percepción del riesgo y la desconfianza hacia los servicios de salud públicos en la incorporación de los CAF en las trayectorias de atención a diversas necesidades de salud?

Con base en estas preguntas, las hipótesis que conducen este trabajo son: a) la pandemia configuró otra relación con los servicios de salud privados y, en particular, con estos consultorios, los cuales ampliaron su abanico de funciones al sumar otras problemáticas de salud que no pudieron ser atendidas por las instituciones públicas; y b) la adhesión de los CAF a trayectorias de atención de personas con distintas necesidades en salud, entre ellas, covid-19, se vio influido por una desconfianza y una percepción del riesgo hacia los servicios de salud públicos, que se agudizó con una crisis sanitaria de esta índole.

A lo largo del trabajo se emplean categorías teóricas como percepción del riesgo y conductas de búsqueda de atención. La percepción del riesgo es un concepto que, en antropología médica, se emplea para comprender cómo las personas entienden el riesgo -de enfermar, de morir, de contagiarse por un virus o bacteria-, y cuáles son los aspectos sociales, culturales o económico-políticos que influyen en la percepción y manejo del mismo. Además, se interesa por estudiar las conductas que los sujetos llevan a cabo para reducir o prevenir el riesgo, denominadas como prácticas de reducción del daño, esto es, formas de “intervenir activamente en la disminución de la sensación de vulnerabilidad y el aumento de la sensación de control del riesgo”^12^. En este trabajo, la percepción del riesgo es una categoría importante que configura las trayectorias de atención de las personas usuarias y la práctica médica en el marco de la pandemia, al igual que la relación con las instituciones de salud.

Igualmente, se incluye el concepto de conducta de búsqueda de atención para ilustrar cómo son conformadas las respuestas sociales al covid-19 y otros problemas de salud, desde el punto de vista de las personas usuarias y de médicos y médicas de los CAF. Este concepto pretende identificar las secuencias de acciones que llevan a cabo las personas para resolver un problema de enfermedad. En la antropología y la sociología médica se han desarrollado varias formas de abordarlo, una de ellas es la trayectoria de atención, retomada en este trabajo, y definida como una categoría teórico-metodológica para analizar la “secuencia de decisiones y estrategias que son instrumentadas por los sujetos para hacer frente a un episodio concreto de padecer”^13^. 

En un contexto de pandemia, las trayectorias de atención de las personas usuarias se ven atravesadas por la percepción del riesgo, el miedo al contagio y la desconfianza hacia los servicios de salud públicos. Como plantea Hevia^14^, la confianza -o desconfianza- es un elemento fundamental para tomar decisiones sobre el sistema de salud a utilizar en caso de tener alguna enfermedad. Siguiendo la definición de confianza que plantea este autor, es decir, como un aspecto que, frente a la incertidumbre del futuro, “aparece como un mecanismo que nos brinda seguridad y tranquilidad (y por ende), nos sentimos menos vulnerables”^14^, se incorpora como un componente que está en juego en situaciones de vulnerabilidad e incertidumbre, como lo es padecer una enfermedad^14^. Por esta razón, la confianza/desconfianza, en conjunto con la percepción del riesgo, opera como un elemento estructural a las secuencias de decisiones y acciones llevadas a cabo por los sujetos en una coyuntura crítica ocasionada por la pandemia, en la que predomina la incertidumbre y la vulnerabilidad.

El artículo se estructura con un primer apartado en el cual se introduce al lector a un panorama sobre el estado de los CAF a nivel nacional y estatal, así como a indicadores sobre su presencia en el contexto de la pandemia. Luego se desarrolla la estrategia metodológica y, posteriormente, los resultados, en los cuales se expone cómo los CAF se situaron como frente de atención al covid-19 y a otras causas; y se describen los factores que influyeron en su utilización. Por último, se expone una discusión que, por un lado, reflexiona sobre las funciones de los consultorios de farmacia en el paisaje pandémico, sus posibles transformaciones y su incorporación a trayectorias de atención de algunas personas usuarias; y, por otro, se problematiza si esta coyuntura da lugar a una reconfiguración de las percepciones y prácticas en torno a la medicina privada y la medicina pública.

### CAF y covid-19 en Oaxaca de Juárez

El surgimiento de los CAF tuvo lugar en la década de 1990, aunque es recién en 2010 cuando se produjo su mayor expansión en todas las entidades del país. A la fecha, según datos de Anafarmex, se estima la existencia de 18.000 CAF^15^. Cabe precisar que, debido a la carencia de datos oficiales y georreferenciados, no se sabe con certeza cuántos hay. De hecho, esta estimación, que proviene de las asociaciones de farmacias, discrepa con las estadísticas proporcionadas por la Secretaría de Salud que, para agosto de 2022, registraban 7.749 CAF^16^, dato obtenido con base en los establecimientos de salud que tienen un registro de clave única (CLUES) en la Secretaría de Salud. Esta divergencia en las estimaciones evidencia tanto una falta de regulación del sector salud, como una invisibilización de estos consultorios expresada en la falta de estadísticas oficiales.

En un estudio realizado por Colchero *et al*.^11^ en el que se analizan los resultados de las Ensanut 2012 y 2018, se muestra un cambio en la oferta de los CAF: se registra un aumento de los CAF relacionado con un menor uso de servicios de salud públicos, incluso entre derechohabientes; así como una modificación en la utilización de los servicios de salud ambulatorios. Sin embargo, ya antes de la pandemia se proyectaba esta tendencia. López Manning y García Díaz^17^ señalan que los CAF son una opción ampliamente aceptada en la búsqueda de atención ambulatoria entre población de diferentes niveles socioeconómicos.

De acuerdo con fuentes de las asociaciones farmacéuticas, los CAF ofrecen un estimado de 10 millones de consultas al mes^15^, lo cual, de acuerdo a una estimación propia, representa el 35% de las consultas generales anuales a nivel nacional^18^^,^^19^^,^^20^. En la coyuntura de la pandemia, los resultados de la Ensanut 2020 sobre Covid-19 indicaron que, sin importar la condición de derechohabiencia, el 23% de las personas con síntomas de covid-19 fueron a consultorios de farmacias^21^; mientras que para 2021, el porcentaje bajó al 11,7%^9^, hecho que probablemente se deba a un subreporte de esta encuesta sobre el uso de los CAF.

Por otro lado, es pertinente analizar los efectos de la pandemia en una entidad como Oaxaca, con mayor vulnerabilidad a una crisis de salud, debido a sus altos índices de pobreza y rezago social, a la precariedad de su estructura sanitaria y a los bajos indicadores en salud^22^. A su vez, es una entidad con un amplio porcentaje de población sin seguridad social, pues solo el 31,7% declaró ser derechohabiente de alguna institución pública^23^.

En los últimos años, al igual que en el resto del país, este municipio junto con los que conforman la Zona Metropolitana de Oaxaca han sido testigos de un crecimiento en la oferta de servicios de salud privados, sobre todo, de establecimientos de atención primaria como los CAF^24^. Se estima que, en 2017, en la entidad federativa, había 1.398 farmacias^25^, 248 con consultorio anexo (16%)^26^. No obstante, este dato también puede presentar un subregistro, sobre todo si consideramos que a nivel nacional el 69,2% de las farmacias que operan en el mercado privado tienen consultorio adyacente^27^. Como se describe en el documento, en esta entidad federativa, los CAF representaron una opción significativa para recibir atención médica. Esto adquiere más trascendencia si consideramos que la pandemia generó una desigualdad en el acceso a servicios de salud, la cual tuvo mayor notoriedad en los servicios estatales de salud.

## METODOLOGÍA

En este artículo se presentan hallazgos de un proyecto de investigación cualitativa realizado entre septiembre de 2020 y agosto de 2022 en el municipio de Oaxaca de Juárez. La información que se presenta fue recuperada tanto del trabajo de campo realizado entre noviembre de 2020 y abril de 2021, y de mayo a julio de 2022; como también de una revisión de fuentes secundarias llevada a cabo en octubre de 2021 y de julio a agosto del 2022.

Es pertinente aclarar que cada uno de los períodos de desarrollo de la investigación representan momentos diferentes sobre el curso de la pandemia y las percepciones sociales sobre ella, lo cual no solo influye en la información obtenida, sino en el curso del trabajo de campo. En las primeras fechas se produjo la segunda ola de la pandemia, una de las más críticas en cuanto a mortalidad. Asimismo, aunque ya había iniciado la vacunación en México, solo estaba dirigida a personal de salud y no a población en general. Cabe aclarar que entre dicho personal no se incluyó a los médicos y las médicas de los CAF quienes, a pesar de las movilizaciones y protestas generadas en diferentes ciudades del país, tuvieron que esperar a ser inoculados por su rango de edad. Mientras que, en la segunda temporada de campo, entre mayo y julio de 2022, un amplio porcentaje de la población ya se encontraba vacunada, y los casos de enfermedad se daban con menor severidad. Estos factores influyeron en la percepción del riesgo de los entrevistados, lo cual pudo ser un factor que colaboró en la mayor aceptación de la investigación por parte de los médicos, las médicas y las personas usuarias.

La unidad de estudio consistió en ocho CAF ubicados en cuatro colonias de la zona central del municipio de Oaxaca de Juárez, cuyos nombres no se revelan para resguardar la confidencialidad de los colaboradores. Se trabajó en este municipio por dos razones: es el que concentra el mayor número de casos acumulados por Covid-19 en la entidad; y en él se localizan el 40% de los CAF de Oaxaca^25^.

De acuerdo a la percepción de los médicos, las médicas y las personas usuarias que se entrevistaron, dichas colonias son consideradas de un nivel socioeconómico medio bajo a medio o medio alto, es decir, no son barrios periféricos en una situación de alta marginación. Sin embargo, ello no necesariamente es un indicador del estrato socioeconómico de las personas usuarias, pues una característica de estas colonias es que, a pesar de ser habitacionales, concentran oficinas y comercios que le imprimen una importante dinámica de movilidad a la zona. Asimismo, una de ellas condensa gran parte de la actividad turística de la ciudad. Estos rasgos generan una lógica de desplazamiento que reúne entre las personas usuarias a quienes viven o trabajan en la zona, a transeúntes ocasionales o turistas.

Por su parte, la unidad de análisis comprende al personal médico y personas usuarias de estos consultorios, con quienes se emplearon distintas técnicas de investigación. De forma específica, se entrevistó a 12 médicos y médicas que, con excepción de una que trabajaba en una farmacia independiente, el resto trabajaban en consultorios de una cadena farmacéutica con amplia presencia nacional. Cinco de ellas eran mujeres y siete varones, con edades que comprendían de los 28 a los 70 años. Todos se habían formado en la Universidad Regional del Sureste (URSE) o la Universidad Autónoma Benito Juárez (UABJO), las universidades con mayor tradición en la región en la formación de médicos.

De igual manera, se aplicó un cuestionario a 59 personas usuarias de tres CAF. Entre algunos datos sociodemográficos relevantes se encuentran que la mayoría eran mujeres (73%), menores de 50 años (71%), y originarios de Oaxaca (88%). Respecto a las ocupaciones, el 29% eran empleados de empresas privadas, el 24% trabajaban para instancias del gobierno, y los restantes se dedicaban al hogar, al comercio, al trabajo independiente, entre otros. Por su parte, el 45% contaba con licenciatura, y el 55% era derechohabiente de una institución pública. El elevado número de personas empleadas en la iniciativa privada o en el sector público, así como el porcentaje de alto nivel educativo y condición de derechohabiencia se deben a las características de las zonas donde se hizo el cuestionario, sobre todo una de ellas que aglomera comercios, restaurantes y oficinas.

Como se mencionó, se hicieron entrevistas en profundidad a médicos y médicas, basadas en una guía de entrevista que abarcó los mismos temas en ambos períodos de trabajo de campo, profundizando más en algunos de ellos. Entre los temas comprendidos en la guía se encuentran: la trayectoria formativa y profesional, el rol de los CAF frente al covid-19 y otros problemas de salud, las experiencias de los médicos y las médicas respecto a su práctica clínica en la pandemia, y los cambios identificados en su saber y práctica médica. En mi primera estancia, entrevisté a ocho médicos y médicas y, en la segunda, a seis, dos de las cuales también fueron entrevistadas en 2021. La duración de las entrevistas fue de dos a cinco horas, con excepción de una que fue de una hora, debido a que no fue posible reencontrarse con el médico. Estas se hicieron de forma presencial en todos los casos, menos uno que fue vía telefónica porque la doctora así lo prefirió. Todas las entrevistas fueron audiograbadas y transcritas.

A su vez, se aplicaron 60 cuestionarios a personas usuarias que contacté en las salas de espera de tres CAF, a partir de los cuales se obtuvo información cuantitativa sobre las causas de consulta, la frecuencia de utilización de los CAF, la percepción sobre la atención en estos consultorios y su utilidad durante la pandemia, al igual que datos sobre el perfil sociodemográfico. Si bien a lo largo del artículo se presenta una parte de la información relevada con este instrumento, su mayor aporte fue que permitió hacer entrevistas informales a varias personas usuarias pues, en muchos casos, al concluir el cuestionario, conversábamos con mayor detalle sobre algún tema. En general, estas entrevistas duraban de cinco a quince minutos y fueron registradas en el diario de campo. En diciembre de 2020, se aplicaron diez cuestionarios piloto que no fueron incluidos en la muestra; y de los 60 cuestionarios aplicados, se invalidó uno por no haber sido completado, por lo que la muestra quedó conformada con 59 cuestionarios.

La estrategia de investigación incluyó un relevamiento de fuentes secundarias que permitió obtener datos estadísticos sobre el número de CAF a nivel nacional y estatal, la utilización de los CAF en la pandemia y su papel en la cascada de atención al covid-19; información sobre declaraciones de las autoridades sanitarias con relación a las funciones de estos consultorios; así como referencias bibliográficas en torno al sector privado y los CAF. Entre las fuentes secundarias consultadas se encuentran artículos periodísticos, encuestas nacionales, registros de asociaciones, conferencias de prensa del gobierno federal y artículos académicos.

Sobre el trabajo con fuentes secundarias, es importante señalar algunas limitantes. Por un lado, el acceso a la información sobre el sector privado en salud representa un desafío al estar en resguardo de las empresas y/o fundaciones y no ser de carácter público, razón que me condujo a buscar otros mecanismos de acceso. En tal sentido, la vía empleada consistió en consultar fuentes periodísticas que recuperan declaraciones o entrevistas realizadas a diversos actores (funcionarios del sector salud, representantes de asociaciones de farmacias o directivos de las cadenas de farmacia), en donde se expresa información relevante sobre estos consultorios.

Lo anterior obliga a hacer un ejercicio crítico respecto al uso de dichas fuentes, y de ese modo interpelar los datos producidos. Así, por un lado, se procura entender los documentos hemerográficos, las encuestas o los informes oficiales como una “forma de conocimiento situado [...] [que] tienen mucho que ofrecernos cuando son vistos como una importante forma discursiva a través de la cual la vida cotidiana es narrativizada y las colectividades son imaginadas”^28^; y, por otro, se intenta apelar al contraste de fuentes como una medida para acercarse a las causas de las incompatibilidades o vacíos de la información. 

El análisis de la información obtenida mediante las entrevistas y observaciones se hizo bajo un procedimiento inductivo con el que se identificaron categorías y subcategorías que delimitaron los ejes temáticos a abordar en los productos de la investigación. Entre las principales categorías que emergieron se encuentran: atención médica al covid-19, cambios y continuidades percibidas, alcances y limitantes de los CAF, y percepciones y prácticas de atención de las personas usuarias. Para realizar la codificación y categorización de los datos, se utilizó el software de análisis cualitativo Atlas Ti, el cual permitió organizar el material en unidades manejables para el análisis. Luego, se realizó el análisis descriptivo de la información, entendido como el “proceso de atribución de significados de datos que ya fueron reducidos y examinados”^29^; para finalmente poder articular el dato científico con el aparato conceptual del trabajo.

En cuanto a las normas éticas de la investigación, en primera instancia, se contó con el consentimiento informado verbal de los médicos, las médicas y las personas usuarias que se entrevistaron, a quienes se les informó sobre los fines de la investigación y el manejo de la información proporcionada. En aras de resguardar el anonimato de los informantes, se emplean pseudónimos y se evita brindar detalles específicos sobre la zona de estudio, como una medida para evitar que estos sean fácilmente identificables.

## RESULTADOS

### De la aplicación de pruebas a la atención al covid-19

Desde el inicio de la pandemia, los CAF asumieron un rol significativo en la atención a personas con síntomas de covid-19. No obstante, una dificultad para visibilizar su envergadura en la atención al coronavirus es que hay pocos datos que nos permitan conocer la cantidad de casos atendidos. De acuerdo a la Ensanut 2021, de las personas diagnosticadas como positivas, el 68% lo hizo en servicios privados, de los cuales el 11,7% ocurrió en farmacias^9^.

A su vez, los registros con los que cuentan las farmacias son poco precisos, lo que dificulta conocer el número real de casos atendidos en estos consultorios. Por ejemplo, en una cadena de farmacias durante la primera etapa de la pandemia, los casos sospechosos y positivos de covid-19 se registraban en el formato del Sistema Único de Información para la Vigilancia Epidemiológica (SUIVE), solicitado por la Secretaría de Salud, como enfermedades respiratorias, y abarcaba todo tipo de afecciones de esta índole, muchas de ellas etiquetadas a nivel emic por los médicos y médicas como “probable covid”. Este término remite a una de las dificultades principales señaladas por los participantes respecto a su quehacer en esta coyuntura, es decir, aprender a diagnosticar una enfermedad nueva en un contexto de difícil acceso a pruebas de detección, como ocurrió en Oaxaca. En la entidad, el sector público no tuvo espacios para realizar pruebas, sino que se hicieron de acuerdo a criterios de priorización. Esto es, se realizaban en unidades de referencia, hospitales y centros de salud de las jurisdicciones sanitarias, pero no a población abierta^30^. 

Lo anterior condujo a que la mayor parte de las pruebas se realizaran en laboratorios privados o farmacias. A finales de 2020, algunas cadenas de farmacias comenzaron a realizar pruebas rápidas de antígeno, tarea que quedó a cargo de médicos y médicas responsables. Por su bajo costo (en comparación a las pruebas PCR que en los laboratorios privados de Oaxaca reportaban un costo superior a $1.800 pesos mexicanos, esto es, aproximadamente $95 dólares americanos) representaron una opción ineludible de acceso a una prueba de detección, como lo indica una usuaria:

*Ayer fui a que me hicieran una prueba a la Farmacia del Ahorro porque empecé a tener dolor de garganta. Por suerte, salió negativa. Yo llegué a las cuatro de la tarde y ya había gente formada, como ya se corrió la voz de que ahí hacen las pruebas, pues está llegando más gente. Pero ayer que fui solo hicieron siete pruebas, nos formaron a los siete y ahí esperamos los resultados. Todos los que estábamos ahí íbamos a eso. Me costó $350, también por eso está llegando la gente, porque está más económica que en otros lugares. Estuve viendo y en otro laboratorio estaba en $1.000, entonces pues sí hay diferencia. Me la tomaron y en 20 minutos me entregaron mi resultado.* (Usuaria 25, diario de campo, 13 de enero de 2021)

Esto no solo implicó una dificultad en el acceso a pruebas diagnósticas, sino que también representó un desafío para el personal médico, al tener que desarrollar criterios diagnósticos basados en datos clínicos. Como se observa en los siguientes fragmentos, las pautas para diagnosticar a pacientes sospechosos se fueron mejorando, lo que da cuenta de cómo la práctica clínica en este contexto se fue transformando por la adquisición de nuevos saberes técnicos sobre la enfermedad, y por la experiencia empírica^31^.

*Al final de cuentas viene siendo una enfermedad nueva, pero ya empiezas a adaptarte y a darte cuenta de la característica de la tos, yo creo que es muy seca. Obviamente, se les pide la prueba, no todos la traen, pero cuando sí, lamentablemente no me he equivocado en la mayoría de los pacientes que resultan positivos a la prueba.* (Dr. Andrés, 17 de diciembre de 2020)

*Entonces la mayoría es más que nada por clínica y porque obviamente empieza uno y ya después sigue el papá, después la mamá, siguen los hijos y obviamente pues es covid, con todos los síntomas, más el contagio que se hizo en la familia, pues casi casi con eso los estamos diagnosticando. Hay algunos que sí se las hacen (las pruebas), pero la mayoría no se la pueden hacer porque están muy caras.* (Dra. Daniela, 11 de abril de 2021)

Otro desafío identificado por los médicos y las médicas consistió en la implementación de esquemas de tratamiento para el coronavirus, sin contar con guías de manejo clínico o protocolos de atención estandarizados. Como se desarrolla en trabajos previos, la adquisición de conocimientos técnico-científicos sobre el virus, su transmisión, evolución y tratamiento, se desarrolló, principalmente, por medios autodidactas, por decisión propia de tomar cursos de actualización y, sobre todo, a partir del conocimiento empírico adquirido al atender pacientes con esta patología^31^.

Bajo esta lógica, los tratamientos prescritos se fueron adecuando más a las recomendaciones internacionales, y se fue haciendo más personalizado, pues los médicos y las médicas detectaron que la sintomatología se presentaba de forma diferente en los pacientes. En tal sentido, en las entrevistas realizadas a finales de 2020, se observó una mayor tendencia a la prescripción de fármacos posteriormente declarados no recomendables:

*Generalmente, manejo algunos antibióticos, aunque sabemos que la cuestión es meramente viral, pero es para evitar la agregación de alguna infección bacteriana. Por ejemplo, la azitromicina, ceftriaxona, algunos pacientes incluso que tienen cierta dificultad respiratoria, el uso de dexametasona, algunos antitusivos, por ejemplo, la levodropropizina, euromicina, benzonatato; medicamentos para el control de la temperatura, principalmente paracetamol; por ejemplo, haciendo uso de este medicamento que se utiliza para la influenza que es el oseltamivir, también he notado que sienten un mejor resultado. También medicamentos anticoagulantes como la aspirina.* (Dr. Andrés, 17 de diciembre de 2020)

En las entrevistas realizadas en 2022 algunos médicos y médicas reconocieron que, cuando la pandemia comenzó, prescribieron medicamentos “no adecuados”:

*El virus ha ido evolucionando. Al inicio se decía que dale ivermectina, que métele hidroxicloroquina o dexametasona. Te soy sincera, yo al inicio receté esos medicamentos ¿por qué? porque tampoco había algo sustentable, se habían hecho algunas pruebas y en algunos pacientes habían funcionado. Yo en mi caso me enfoqué más en la experiencia y en los estudios previos que se tienen de las afecciones respiratorias* […] *Ahora es muy importante la exploración física, si yo te ausculto pulmón y escucho que hay sibilancias, que hay estertores, que hay algo que me esté guiando hacia una inflamación pulmonar o ya como tal una neumonía, pues te enfocas en eso, entonces tu desinflamatorio; si ya escucho que tienes sibilancias pues bueno va tu Salbutamol. Entonces, yo me guie en cuanto a los síntomas que presenta mi paciente, o sea, el tratamiento era como que más individualizado.* (Dra. Carmen, 16 de junio de 2022)

La atención al covid-19 en los CAF también supuso la fase del seguimiento a pacientes. Como ocurrió con otros profesionales médicos, frecuentemente se hizo por medios alternativos: llamadas, mensajes telefónicos o videollamadas; sin embargo, se identificó que en los CAF el seguimiento presencial tuvo un lugar importante, sobre todo, con la finalidad de monitorear la saturación de oxígeno, debido a que no todos los pacientes tenían acceso a un oxímetro. De hecho, a partir de la pandemia esta cadena de farmacia incluyó, en su lista de servicios, la oximetría, por un costo de $20.

Los CAF, como frente de atención al covid-19, desempeñaron otras funciones como fue la valoración de pacientes después de haber padecido esta enfermedad para identificar alguna secuela. Un caso que lo ilustra es el de una señora de 65 años que conocí en la sala de espera de un consultorio, mientras esperaba a ser atendida a diez días de haber sido diagnosticada con el virus. La mujer se había atendido con el médico de su centro de trabajo, pero decidió acudir con la doctora Gema para valorar el estado de sus pulmones, por lo cual llevaba una radiografía de tórax que esta le había solicitado previamente. Por otro lado, se detectó la prescripción de recetas en casos de pacientes que eran atendidos por otros médicos, pero requerían una receta para que se les vendiera el fármaco, o bien, porque no querían salir a buscar atención. Un ejemplo de esto es el de un joven cuyo padre tenía covid-19 y necesitaba que la doctora le recetara un tratamiento, algo que ella no aceptó.

### “*¿Qué opción les queda a las personas, más que nosotros?*”: Reconversión hospitalaria, miedo y desconfianza

Se ha señalado cómo las Ensanut 2020 y 2021 dan cuenta de un amplio porcentaje de personas que acudió a los servicios de salud privados para atenderse por síntomas de covid-19. Por su parte, datos obtenidos mediante un cuestionario que apliqué a 59 personas usuarias de CAF, entre los meses de enero a abril de 2021 y de mayo a julio de 2022, muestran que el 66% acudiría a este servicio en caso de presentar síntomas de covid-19. Aunque no es una muestra representativa, sí resulta un dato que puede funcionar como un indicador que da cuenta de hipotéticas prácticas de atención ante un evento de covid-19. Empero, más allá de referir a porcentajes, el objetivo de este apartado es presentar los principales factores que influyeron en la utilización de los CAF durante la pandemia.

Con la finalidad de asegurar el acceso oportuno y la calidad de la atención a pacientes que presentaron una infección de covid-19, el gobierno federal implementó la estrategia de reconversión hospitalaria, que buscó ampliar la capacidad de respuesta y organización de los servicios de segundo y tercer nivel^5^. Aunque esta permitió atender a una mayor cantidad de pacientes con cuadros moderados a graves, tuvo consecuencias negativas en el acceso a los servicios de salud. En un análisis realizado por el Centro de Investigación Económica y Presupuestaria, se encontró que unas 42,2 millones de consultas dejaron de hacerse durante 2020, lo que significó una caída del 48,6% respecto a las realizadas en 2019^32^.

A esto debe sumarse que la pandemia propició otras necesidades de salud. Como mencionan los médicos y las médicas que se entrevistaron, la pandemia modificó las causas de consulta, por lo que aumentaron las consultas por enfermedades crónico-degenerativas y por problemas de salud mental como ansiedad o depresión. De acuerdo a sus comentarios, la atención a enfermedades crónicas se incrementó debido a que varias personas con estas condiciones dejaron de ser atendidas en las instituciones públicas, a causa de la reconversión hospitalaria o, como sucedió en esta entidad, por el cierre de unidades médicas. Asimismo, el incremento también se debió a que, como consecuencia del covid-19, algunas personas desarrollaron una enfermedad de esta índole:

*Aumentó mucho la consulta de pacientes crónicos porque, precisamente, no los atendían ni en el IMSS* [Instituto Mexicano del Seguro Social]*, ni en ningún otro lado pues, entonces iban ahí* [al consultorio]*, y ahí llevábamos nosotros su tratamiento, incluso muchos se quedaron con nosotros. Aumentó bastante, sobre todo los diabéticos e hipertensos* […] *A algunos se les da seguimiento, yo tengo pacientes que van cada mes para ver cómo están sus niveles. Tengo varios que tuvieron covid y que ahora les llevo su control de enfermedades crónicas, o sea, ahora sí estamos viendo todas las complicaciones que les pudo haber dejado el covid.* (Dra. Daniela, 26 de mayo de 2022)

El cierre temporal de unidades médicas públicas, principalmente de atención primaria, fue un factor que influyó en la búsqueda de atención en los CAF por parte de personas usuarias que anteriormente habrían acudido al centro de salud:

*Ahora en la pandemia todos los centros de salud cerraron, en lugar de ayudar a la gente en la pandemia, todos estaban cerrados y la gente tenía que acudir con nosotros porque no encontraban nadie más. Incluso hay muchos hospitales, muchos centros de salud que tenían al paciente con una prueba positiva de covid y no los atendían, entonces tenían que venir con nosotros y realmente la consulta es muy barata para tanto riesgo. Entonces yo sí creo que los consultorios han ayudado mucho ¡a mucha gente! Más aquí en Oaxaca, más en esos momentos.* (Dra. Daniela, 19 de mayo de 2022)

*Yo considero que sí fuimos importantes, no que seamos imprescindibles pero sí somos el primer contacto, o sea, los centros de salud dejaron de laborar, yo tengo ahí un SESA* [Servicios Estatales de Salud] *en la misma comunidad, dejó de laborar, no atendía nada, el SESA de ahí es ginecológico, bueno como todos los que pusieron, entonces, solamente embarazadas veía. A los crónicos degenerativos los hicieron a un lado, urgencias para nada, respiratorios no querían ver* […] *El personal del centro de salud se ausentó, tengo una conocida que es enfermera, me dice “es que se fueron, no han regresado, ya están vacunados y no han regresado”. Entonces también es irresponsabilidad, y pues ¿qué opción les queda a las personas, más que nosotros?* (Dra. Gema, 4 de abril de 2021)

Esta situación hizo que los CAF representaran una opción sustancial para recibir atención primaria. Como lo señala una usuaria, una de las mayores ventajas es que nunca dejaron de atender pacientes, a diferencia de otros lugares:

*La verdad sí creo que estos consultorios ayudaron mucho porque estuvieron abiertos toda la pandemia, nunca cerraron, era de lo poco que quedó abierto porque ya ves que casi en todos lados dejaron de dar servicio.* (Usuaria 6, diario de campo, 8 de junio de 2022)

Desde el comienzo de la pandemia, los CAF brindaron atención a todos los pacientes, incluso a aquellos que acudían con síntomas respiratorios. Pese al temor y, en algunos casos, reticencias de las y los profesionales, se les siguió atendiendo por indicación de los directivos de la cadena de farmacia. De hecho, para evitar que los médicos y médicas rechazaran a estos pacientes, se colocaron letreros en las salas de espera con la frase “*en este consultorio se atienden enfermedades respiratorias*”. Por esto, siguieron atendiendo una de las principales causas de consulta en los CAF, las enfermedades respiratorias^33^^,^^34^. A pesar de la pandemia, esta continuó siendo la principal causa de consulta, como fue referido por las personas usuarias entrevistadas. De estos, casi la cuarta parte fueron por ese motivo (23,7%).

Al respecto, se advirtió que personas con síntomas respiratorios -que probablemente antes de la pandemia no habrían acudido al médico y se habrían automedicado- buscaron atención médica, por miedo a que se tratara de covid-19:

*He venido muchas veces porque por mi ansiedad he creído que tengo covid, empiezo a sentir que no puedo respirar o que me duele la garganta, y vengo, me revisan y me dicen que estoy bien, que sólo es infección de anginas que es de lo que yo padezco mucho. A veces me mandan a hacer pruebas, y siempre salen negativas. Hay veces en que me he hecho hasta tres pruebas al mes, pero todo es por mi problema de ansiedad. Una vez tuve el oxímetro todo el día porque yo sentía que no podía respirar bien. Ahorita que venía para acá sentí que no respiraba bien, traté de tranquilizarme y ahorita ya estoy bien.* (Usuario 6, diario de campo, 8 de junio de 2022)

*Yo traigo el cubrebocas todo el día, pero lo que siento es que me está dando alergia porque tengo mucho flujo, me escurre mucho la nariz. Yo pienso que es por el cubrebocas, y eso que siempre que llego a mi casa en las noches lo lavo y al día siguiente me pongo otro. Y ahorita vengo por eso, para que me revisen, como dicen que ahorita no hay que automedicarse. Normalmente, yo no vengo a los médicos, por eso te digo que esta es la primera vez que vengo a uno de estos, yo lo que hago es que me aguanto. Cuando me da gripe, me aguanto y me aguanto y ya hasta que estoy muy mal voy al médico. Luego lo que me tomo es el Rosel, que es muy bueno, pero ahorita por eso de que no es bueno automedicarse preferí venir a que me checaran, que igual no creo que sea covid porque ya ves que los síntomas son otros, y ahorita nada más traigo el escurrimiento.* (Usuaria 39, diario de campo, 1 de marzo de 2021)

Otro factor determinante en la utilización de los CAF consistió en la reproducción de una percepción sobre los hospitales, en tanto lugares de mayor riesgo de contagio, por ser espacios donde se atendía a personas con covid-19 y que presentaron una saturación importante. De ese modo, en la percepción de las personas usuarias, el consultorio de farmacia se configuró como un espacio más “seguro”, como lo señalaron el 20% de los encuestados, al preguntarles sobre los motivos por los cuales preferirían acudir a un CAF en caso de presentar síntomas de covid-19:

*Preferí venir acá porque es más económico, y además los hospitales estaban abarrotados de gente que estaba enferma. Y la verdad es que aquí te atienden mejor que en tu propia institución de salud, en mi caso yo tengo IMSS.* (Usuario 11, diario de campo, 13 de junio de 2022)

*Estos consultorios ayudaron bastante porque los servicios de salud, como el seguro social, el Hospital Civil y los centros de salud, estuvieron abarrotados. No era confiable llevar a mis hijos ahí, y aquí es como más fluida la atención, entonces siento que es más confiable.* (Usuaria 28, diario de campo, 2 de julio de 2022)

*De hecho, ya tuve y me estuve tratando en un consultorio de farmacia, no en este, en otro que me recomendaron. Tengo IMSS, pero preferí venir aquí porque una amiga ya se había tratado con ese doctor y me lo recomendó, pero más que nada porque* sí siento que en el IMSS hay más riesgo, me siento más segura aquí. Fue por eso*.* (Usuaria 41, diario de campo, 30 de marzo de 2021)

Estos testimonios son concordantes con los publicados en una encuesta realizada a mediados de 2020 por un medio de prensa nacional, donde se calculó que casi el 70% de los encuestados se sentirían inseguros si llevaran a sus seres queridos a un hospital durante la pandemia^35^. Además, la percepción sobre los hospitales como espacios de riesgo de contagio que generan desconfianza se debe también a su asociación con lugares de riesgo de muerte. Al respecto, médicos y médicas señalan que, a pesar de referir a los pacientes al segundo o tercer nivel, eran pocos los que efectivamente seguían esta recomendación:

*Yo le dije a la paciente “sería bueno que lo llevara a un hospital, solo como revisión”, yo no le dije que lo internen, pero ellos no van porque sienten ese miedo, un tanto porque te internan y ya no sales; otro tanto, la gente se espera y ya llega con las personas muy mal, prácticamente muertos, entonces también la gente del hospital ya no los recibe, no sé por qué, tal vez porque no hay camas o tal vez les da miedo también, los ven ya tan mal que no les abren la puerta.* (Dr. Jacinto, 20 de febrero de 2021)

*Fuimos este año la mejor opción para la gente de no irse a algún hospital. Las personas que llegaban me decían “no, es que yo no quiero ir al hospital”, era un terror, o es un terror, que tú les digas “¿tiene usted institución? Tiene que ir al seguro”, “no, es que yo no quiero ir”. Les da miedo ir por el contagio, y porque como van platicando, se van pasando de boca en boca la información, entonces les dicen “no es que si tú llegas con una cuestión respiratoria te van a ingresar, y ya no sales ¡eh!” y “¡ahí te mueres!” Entonces la información mala o el teléfono descompuesto, hace el temor.* (Dra. Sandra, 4 de abril de 2021)

Hasta ahora se describieron los principales motivos por los cuales los CAF fueron un recurso de atención preponderante en la pandemia; sin embargo, es importante señalar dos cuestiones. La primera es que la percepción de las personas usuarias sobre la atención recibida es diversa y crítica respecto a sus limitantes. De las 39 personas que afirmaron acudir a un CAF en caso de tener síntomas de covid-19, el 25% aseguró que lo haría solo en caso de presentar un cuadro leve, o bien, como una segunda opción en caso de no poder asistir a un médico particular, la alternativa más idónea. Precisamente, de las 16 personas cuyas respuestas fueron que no irían a un CAF en caso de covid-19, el 44% preferiría ir con un médico particular, por asumir que se trata de una enfermedad que requiere una atención más especializada, que un médico de farmacia no puede proveer.

La segunda cuestión es que para entender la configuración de la búsqueda de atención al covid-19 y el papel que en ello tienen los consultorios, hay que considerar el diagnóstico que la persona y su grupo familiar hace sobre la gravedad de su cuadro. En tal sentido, generalmente se trataba de personas con cuadros leves a moderados y, solo en algunos casos eran personas graves que no podían acceder a ningún otro recurso y que, frecuentemente, ya no tenían nada que perder en su trayectoria de atención, como lo narra la doctora Carmen al referirse a un paciente grave que atendió a domicilio a petición de sus familiares:

*Varios murieron en casa, me tocó un pacientito que ya había sido internado en el hospital, estaba con una neumonía grave, pero cuando le dijeron que tenía que entubarse, él dijo que no y le dijo a su familia que lo sacaran, que no quería. Se regresó a su casa, al siguiente fue cuando sus familiares me hablaron, me dijeron “doctora, es que quiero que vaya a ver a mi papá porque no puede respirar, se está poniendo morado y no sabemos qué hacer”. Pues bueno, vamos a ver cómo está el señor. Fuimos a revisarlo, pero desafortunadamente no sobrevivió ni a la noche, o sea, ya de plano no se podía, estaba como a veinticinco litros por minuto, con dos tanques de oxígeno, tenía el concentrador aparte. Él decidió morirse en su casa y varios así me enteré que fallecieron en su domicilio.* (Dra. Carmen, 11 de junio de 2022)

## DISCUSIÓN

La discusión sobre los principales hallazgos se organiza en tres ejes: las funciones de estos consultorios en el marco de la pandemia; los factores que influyeron en la utilización de los CAF durante la pandemia; y el posicionamiento de “lo privado” en las percepciones y prácticas de atención a la salud.

La pandemia configuró un contexto particular a nivel del sistema sanitario mexicano, pues este se encontraba en una crisis devenida de las políticas de salud de las últimas décadas, que afectaron particularmente la atención primaria, en un momento de transición al Instituto de Salud para el Bienestar (INSABI), y de fuertes polarizaciones y críticas hacia las estrategias implementadas por las autoridades sanitarias a la llegada del coronavirus al país. En esta coyuntura, los CAF ya representaban un componente medular del sistema de salud en México, como lo muestran diversos estudios^17^^,^^33^^,^^36^^,^^37^. No obstante, en el marco de una crisis sanitaria que obligó a modificar temporalmente la estructura sanitaria, con la estrategia de reconversión hospitalaria, el cierre de unidades médicas o la suspensión de servicios a la población en general, y la saturación del sistema público de salud, mucha gente se vio obligada a buscar atención en otros lugares. Si bien otros estudios como el realizado por Colchero, Moreno y Bautista^21^ ya han dado cuenta de la relevancia que tuvieron los CAF en la cascada de atención al covid-19, en el presente artículo se ha buscado refrendar este planteamiento desde un enfoque cualitativo que recoge la perspectiva del personal médico y de las personas usuarias.

De ese modo, entre las principales funciones de estos consultorios como frente de atención al covid-19, se encuentran su labor diagnóstica al aplicar pruebas rápidas a un precio más accesible que los laboratorios privados; y el haber sido centros de atención a personas con síntomas de covid-19 que no querían acudir a instituciones públicas, o eran rechazadas de ellas, y que tampoco podían pagar una consulta con un especialista privado. Aunque en su mayoría se trató de personas con cuadros leves de coronavirus, también hubo casos de gravedad que no tuvieron otra opción para recibir atención. A esto podemos añadir que en estos consultorios se hicieron valoraciones poscovid-19 a los pacientes, y se trató a quienes presentaron alguna secuela.

Sin embargo, sus funciones no se limitaron a la atención al covid-19. En los CAF “*la vida siguió*”, médicos y médicas no dejaron de trabajar -pese a las reticencias y el temor por parte de algunos- y siguieron dando atención a todos las personas. Así, la gente continuó llegando por infecciones gastrointestinales, urinarias, dermatitis, lesiones articulares y musculares, entre muchas otras causas. Una parte importante fueron enfermedades respiratorias que no eran covid-19, y que sobre todo al inicio -cuando el saber médico al respecto aún era limitado y se acompañaba de incertidumbre y miedo de diagnosticar y tratar a las personas- continuaron siendo valoradas presencialmente, contrario a otros servicios médicos. Igualmente relevante fue la atención que se dio a enfermedades que presentaron una mayor demanda, como las enfermedades crónicas y los trastornos de salud mental. En este punto, resulta imprescindible que otros trabajos inquieran cómo la pandemia modificó las causas de consulta en la atención primaria y especializada, generando otras necesidades de salud que podrían tener implicaciones en la demanda de servicios sanitarios a corto y mediano plazo. 

Todo esto ocurrió en un contexto dinámico marcado por la pandemia, lo que supuso desafíos para el personal médico, el cual tuvo que modificar ciertos aspectos de su ejercicio profesional. En un trabajo previo expuse cómo se configuró la percepción del riesgo en médicos y médicas, y de qué forma influyeron aspectos como su condición de vulnerabilidad o las conductas de rol definidas por la profesión médica^30^. En el presente documento, se retrató la necesidad de adaptar el saber médico a las nuevas circunstancias, como ocurrió con las pautas de diagnóstico y las prescripciones.

El segundo eje de discusión alude a los factores que influyeron en la búsqueda de atención al covid-19 u otras enfermedades. Primero que nada, debe entenderse que estos factores se suman a los ya revelados antes de la pandemia. En otra investigación encontré que la utilización de los CAF varía en función de la gravedad percibida del padecimiento, o de quién sea el paciente. Por ejemplo, frecuentemente, a los niños pequeños se prefiere llevarlos a un médico particular o a un pediatra^34^. En el presente caso, tales criterios son igualmente operativos, es decir, el criterio de gravedad es fundamental en la decisión de acudir a un CAF, percibidos como lugares donde solo se atienden enfermedades leves; o bien, el perfil del paciente, pues generalmente eran personas adultas en edad productiva quienes acudían, pero escasamente adultos mayores, considerados como personas con mayor riesgo de desarrollar un cuadro de gravedad.

Al identificar estos criterios, entran en juego otros factores que configuran la trayectoria de atención: a) el papel que desempeñan los servicios de salud públicos ambulatorios en las experiencias y saberes de las personas usuarias; b) la accesibilidad física, económica y administrativa que caracteriza a dichos espacios; c) la percepción sobre la atención médica recibida; y d) sobre la eficacia terapéutica^34^. En el caso aquí analizado, estos factores se añaden a los que devinieron de la pandemia: por un lado, el cierre de unidades médicas y la suspensión de la atención en los hospitales reconvertidos, al igual que la saturación de las clínicas y hospitales públicos; y, por otro, la percepción del riesgo orientado en dos direcciones: el contagio y la muerte. Es así que acudir a los CAF se convirtió en una práctica de reducción del daño que las personas usuarias llevaron a cabo para disminuir la sensación de vulnerabilidad y aumentar la sensación de control del riesgo^12^.

En esa línea, la desconfianza hacia los servicios de salud públicos se sitúa como un elemento que se agudiza con esta coyuntura, algo que ha sido abordado por otros trabajos, al mostrar cómo este comportamiento también se expresó en otros brotes epidémicos recientes como la influenza AH1N1 o el zika^38^^,^^39^. Bajo este sentido, es pertinente argumentar que acontecimientos de este tipo pueden ser determinantes para una creciente erosión de la confianza en el sistema de salud público, y quizá un mayor distanciamiento sociocultural entre ciudadanos, la autoridad y las instituciones del Estado^40^, algo que será necesario indagar por investigaciones con otros enfoques metodológicos.

Por último, se discute en torno a cómo la pandemia produjo una recaracterización y, posiblemente, una resignificación del sector privado en salud. Al igual que el sistema de salud público, el sistema de salud privado en México está segmentado, diversificado y poco regulado. Si bien en las últimas décadas se produjo una mayor privatización de la salud, la evidencia empírica indica que con la pandemia este fenómeno se magnificó. Ello no solo modifica la organización del sistema sanitario mexicano, sino también las concepciones y prácticas de las personas en torno a lo público y lo privado, particularmente, en el primer nivel de atención que es en donde se situó la presente investigación.

La valoración positiva sobre los servicios de salud privados es algo que ha sido documentado reiteradamente en diversos estudios antropológicos^2^. En un trabajo realizado por Osorio con personas usuarias de los CAF en la Ciudad de México, se encontró que a pesar de las evaluaciones positivas sobre los CAF, los médicos privados eran vistos como el recurso que provee mejor atención y donde se prescriben medicamentos más eficaces^41^. Durante la pandemia, se reforzó la percepción positiva sobre los servicios privados -ya sean CAF, consultorios de médicos generales, clínicas u hospitales- como lugares en los cuales es más probable curarse o, simplemente, no morir. A partir de esta posible resignificación de lo privado que necesita seguir siendo explorada, es indispensable estudiar qué implicaciones puede tener para la salud y la economía de la población, y así provocar un aumento del gasto de bolsillo o un mayor consumo de fármacos.

En este panorama, se aprecia que, ante la incapacidad del estado para proveer atención médica en una situación crítica, el modelo de atención privado de los CAF, asentado en los últimos años como uno de los principales proveedores de atención primaria en contextos urbanos, tuvo una exitosa inserción en las prácticas de atención de personas con amplias necesidades en salud. Esto no implica asumir una postura favorable hacia este modelo, sino más bien enfatizar en la necesidad de generar distintos análisis sobre cómo dicha modalidad de privatización de la salud se adapta a las necesidades sanitarias de los contextos locales que, a su vez, pueden producir repercusiones negativas para las poblaciones y los trabajadores sanitarios, entre las cuales se encuentran la sobreprescripción de medicamentos, las iatrogenias médicas, el incremento del gasto de bolsillo, o la precarización y degradación de la profesión médica^42^. 

## REFLEXIONES FINALES

La información presentada muestra que los CAF continuaron desempeñando las funciones que ya cumplían desde antes de la pandemia, pero sumaron otras más, como el diagnóstico y la atención al covid-19, lo que los posiciona como una pieza clave del sistema de salud mexicano. Ahora bien, pese a ello predomina un desconocimiento y una desestimación por parte de ciertos actores del sector salud oficial, como se pudo advertir en las declaraciones emitidas por un funcionario de salud al etiquetarlos como “aventuras comerciales” o lugares que solo buscan hacer negocio, por lo cual lo ideal sería desmantelarlos^43^^,^^44^.

Precisamente, la pandemia hizo necesario voltear a ver los puntos ciegos del sistema de salud. En esta tarea, contar con abordajes cualitativos y etnográficos que documenten qué hizo la gente para atender su salud es fundamental. Desde luego que esto no solo incluye el análisis de los recursos biomédicos, sino también de otros saberes y formas de atención, la medicina tradicional y alternativa, o la autoatención, todos ellos puntos ciegos del sistema sanitario. Por ende, uno de los principales aportes del presente trabajo es poder visibilizar, por un lado, algunas prácticas de atención llevadas a cabo por los sujetos durante la pandemia; y, por otro, plasmar las múltiples y complejas funciones desempeñadas por los CAF en este momento de crisis.

A lo largo del artículo, se identificaron algunas vetas de estudio. Una de ellas refiere a un hallazgo indirecto de esta investigación, es decir, el incremento de la medicalización expresada en distintos niveles: la incorporación del curador especializado en episodios de gripes que anteriormente se habrían resuelto en el ámbito de la autoatención; la integración de nuevos insumos a la vida cotidiana como el cubrebocas, el oxímetro, el termómetro digital, etc.; o el aumento de otras condiciones de salud que requieren intervención médica, como problemas de salud mental. Por ello, es necesario investigar a profundidad en qué medida un episodio que irrumpió en el funcionamiento de los sistemas de salud, pero también en las representaciones y prácticas de los sujetos sociales en torno a la salud y la enfermedad, como lo es la llegada del covid-19 al mundo, produjo nuevas prácticas de medicalización, entendida como el proceso mediante el cual se define -o redefine- un problema en términos médicos, y que requiere de una intervención médica para tratarlo^45^.

Una segunda veta consiste en la importancia de generar investigaciones cualitativas que indaguen en la percepción social sobre la medicina privada, pues los sistemas clasificatorios legos sobre este aspecto son complejos debido a la heterogeneidad del sector privado. De hecho, los CAF no suelen ser concebidos como servicios privados, sino como un recurso con características diferentes a las del médico particular.

A modo de cierre, los CAF llegaron para llenar nichos vacíos del sistema de salud público que, con la pandemia, se situaron como lugares menos riesgosos y más confiables, pero sobre todo como servicios capaces de brindar atención primaria a personas con una amplia gama de necesidades sanitarias. En este panorama, cabe preguntarse qué ocurrirá una vez que la pandemia se convierta en un fenómeno endémico, y el sistema de salud se vaya reajustando a la dinámica prepandémica.
